# Points

**Published:** 1887-02

**Authors:** J. E. Lilienthal

**Affiliations:** New York City


J. E. Lilienthal, M. D., New York City.

In the December number of The Homœopathic Physician the Editor refers to the article of Dr. C. C. Smith under the above heading, and requests readers, knowing of other remedies having the same or similar symptoms, to furnish them for insertion. Wishing to add my mite, I contribute the following:

Taste of rotten eggs. Hepar (Jahr.).

Children are easier when carried about quickly. Verat. alb. (C. Hg.)

Desire for warm drinks. Cascarilla (C. Hg.); Cedron (C. Hg.).

Desire for warm drinks. Eupator. purp. (Butler in Hom. Phys., May, 1883).

Desire for warm drinks. Hypericum (C. Hg.); Medorrhinum.

Better, wrapping up the head and keeping in a dark room. Mezereum (C. Hg.).

The brain seems to shake when walking or running in open air; better when wrapping head up in warm room and when at rest. Nux vom. (C. Hg.).

Colic, relieved by pressure—carrying on mother's shoulder. Cina, Podophyl. (S. L. Therapeutics).

